# Epigenetic Subgroups of Esophageal and Gastric Adenocarcinoma with Differential *GATA5* DNA Methylation Associated with Clinical and Lifestyle Factors

**DOI:** 10.1371/journal.pone.0025985

**Published:** 2011-10-20

**Authors:** Xinhui Wang, Gyeong Hoon Kang, Mihaela Campan, Daniel J. Weisenberger, Tiffany I. Long, Wendy Cozen, Leslie Bernstein, Anna H. Wu, Kimberly D. Siegmund, Darryl Shibata, Peter W. Laird

**Affiliations:** 1 Department of Preventive Medicine, Keck School of Medicine, University of Southern California, Los Angeles, California, United States of America; 2 Department of Surgery, Keck School of Medicine, University of Southern California, Los Angeles, California, United States of America; 3 Department of Pathology, Cancer Research Institute, Seoul National University College of Medicine, Seoul, Korea; 4 Division of Population Sciences, City of Hope National Medical Center, Duarte, California, United States of America; 5 Department of Pathology, Keck School of Medicine, University of Southern California, Los Angeles, California, United States of America; 6 Department of Biochemistry and Molecular Biology, Keck School of Medicine, University of Southern California, Los Angeles, California, United States of America; 7 University of Southern California Epigenome Center and USC/Norris Comprehensive Cancer Center, Los Angeles, California, United States of America; Bellvitge Biomedical Research Institute (IDIBELL), Spain

## Abstract

**Background:**

Adenocarcinomas located near the gastroesophageal junction have unclear etiology and are difficult to classify. We used DNA methylation analysis to identify subtype-specific markers and new subgroups of gastroesophageal adenocarcinomas, and studied their association with epidemiological risk factors and clinical outcomes.

**Methodology/Principal Findings:**

We used logistic regression models and unsupervised hierarchical cluster analysis of 74 DNA methylation markers on 45 tumor samples (44 patients) of esophageal and gastric adenocarcinomas obtained from a population-based case-control study to uncover epigenetic markers and cluster groups of gastroesophageal adenocarcinomas. No distinct epigenetic differences were evident between subtypes of gastric and esophageal cancers. However, we identified two gastroesophageal adenocarcinoma subclusters based on DNA methylation profiles. Group membership was best predicted by GATA5 DNA methylation status. We analyzed the associations between these two epigenetic groups and exposure using logistic regression, and the associations with survival time using Cox regression in a larger set of 317 tumor samples (278 patients). There were more males with esophageal and gastric cardia cancers in Cluster Group 1 characterized by higher GATA5 DNA methylation values (all p<0.05). This group also showed associations of borderline statistical significance with having ever smoked (p-value = 0.07), high body mass index (p-value = 0.06), and symptoms of gastroesophageal reflux (p-value = 0.07). Subjects in cluster Group 1 showed better survival than those in Group 2 after adjusting for tumor differentiation grade, but this was not found to be independent of tumor stage.

**Conclusions/Significance:**

DNA methylation profiling can be used in population-based studies to identify epigenetic subclasses of gastroesophageal adenocarcinomas and class-specific DNA methylation markers that can be linked to epidemiological data and clinical outcome. Two new epigenetic subgroups of gastroesophageal adenocarcinomas were identified that differ to some extent in their survival rates, risk factors of exposure, and *GATA5* DNA methylation.

## Introduction

Promoter CpG island hypermethylation is a common occurrence in human cancers, and is generally associated with transcriptional silencing of the associated gene [1], [2]. Although much of the focus has been on tumor-suppressor genes silenced by promoter hypermethylation, many CpG island hypermethylation events occur either outside of promoters, or at genes without a known role in cancer, or even at genes normally not expressed in that particular cell lineage. Thus, many CpG island hypermethylation events are likely to reflect passenger events, rather than drivers of the oncogenic process. We and others have shown that targets for transcription repression by the Polycomb group (PcG) proteins in human embryonic stem cells are particularly predisposed to CpG island hypermethylation in cancer [3], [4], [5]. Irrespective of their role in the oncogenic process, all cancer-specific DNA methylation changes constitute potential biomarkers that might be exploited as clinical tools for diagnosis or early detection of cancer, appraisal of disease progression or response to therapy, or risk assessment in surveillance programs [6].

Esophageal adenocarcinoma (EAC) is a very aggressive disease that metastasizes early and has a poor prognosis with 5-year survival rates less than 40% [7]. For reasons that are not well understood, the incidence of this type of cancer has increased in the past three decades in the Western industrialized countries especially among white males [8], [9]. Gastroesophageal reflux disease (GERD) is a major risk factor for this type of cancer [10]. EAC arises from the epithelial lining of the distal esophagus, particularly when this mucosa has undergone metaplastic change, characteristic of Barrett's esophagus. The epithelium of the gastroesophageal junction shares histological characteristics with distal esophageal mucosa, and is quite distinct from epithelium lining the gastric cardia (proximal stomach) or distal regions of the stomach. However, tumors arising in the gastric cardia share more clinical, epidemiological and molecular features with EAC than with tumors arising in the distal part of the stomach. Moreover, while the incidence of distal gastric adenocarcinoma (DGA) has decreased dramatically during the past five decades, the incidence of gastric cardia adenocarcinoma (GCA) has increased slightly especially among men in Western countries and then remained relatively stable since 1990 [11], [12]. This suggests that the reflux-associated EAC and adenocarcinoma of the gastric cardia and gastroesophageal junction could be considered to be etiologically related and possibly reflective of a similar disease process [13], [14]. However, many clinicians view these diseases as distinct entities that would require better means than pathological evaluation for their classification [15].

We have previously shown that DNA methylation profiles differ between normal squamous esophageal mucosa and the very early precursor lesions of EAC, Barrett's intestinal metaplasia, and dysplasia [16], [17]. We have also shown that hypermethylation of the *APC* gene promoter in the plasma of patients with EAC is associated with reduced patient survival [18]. Since then, more groups have reported other DNA methylation abnormalities in the gastroesophageal cancers [19], [20], [21]. However, there are only few reports in which large numbers of tumor samples and markers have been used in epigenetic profiling experiments or to identify the associations of epigenetic alterations with clinical pathological and lifestyle risk factor data.

In this study we performed a large-scale DNA methylation analysis on a subset of 318 tumor samples (279 patients) of esophageal and gastric adenocarcinomas obtained from a multi-ethnic population-base case-control study using a total of 79 DNA methylation markers. The main goal of the study was to identify markers that distinguish the different types of cancer in the esophageal-gastric continuum, as well to identify novel subgroups of gastroesophageal cancers based on their DNA methylation profiles and to assess their association with known epidemiological risk factors and clinical outcomes. This study was also intended to explore the possibility of using DNA methylation analysis to conduct future population-based studies that typically have limited amounts of paraffin-embedded tissues specimens, but have the advantage of availability of extensive clinical/pathological and lifestyle risk factor data.

## Materials and Methods

### Ethics Statement

This study was conducted in accordance with the Helsinki human subjects doctrine and was approved by the Institutional Review Board of the Keck School of Medicine of the University of Southern California. Signed informed consent was obtained from all study participants for the collection of the samples and their subsequent analysis.

### Specimen collection and selection for methylation and statistical analyses

Patients included in this study were cases from a population-based case-control study of esophageal and gastric adenocarcinoma we conducted in Los Angeles County [22]. In brief, histologically confirmed esophageal or gastric adenocarcionomas were identified from the NCI Surveillance, Epidemiology and End-Results Program (SEER) population-based cancer registry covering Los Angeles County, the USC Cancer Surveillance Program (CSP). A total of 942 patients (222 esophageal adenocarcinomas, 277 gastric cardia adenocarcinomas, and 443 distal gastric adenocarcinomas) were interviewed and 879 signed a medical release giving us permission to obtain their tumor samples from the hospital of diagnosis. Tumor blocks were obtained on 542 patients but the amount of tumor tissue was too small or degraded for 162 patients and thus DNA material was available on 380 patients (97 esophageal adenocarcinomas, 105 gastric cardia adenocarcinomas, 178 distal gastric adenocarcinomas). We obtained 523 formalin-fixed paraffin-embedded slides from 380 patients but had to further exclude another 83 slides after prescreening the samples for DNA quantity. We selected only the 332 tumor samples (292 patients) from the remaining 440 samples to perform the DNA methylation analysis. We further excluded 14 samples (13 patients): one sample was from a patient that had previous cancer, eight samples did not have gastric/esophageal tissues, and five samples were omitted because the reported daily caloric intake of these patients was either too low (≤500 calories per day) or too high (>7,000 calories per day). The remaining 318 tumor samples (279 patients) were used in the DNA methylation analysis. Forty five of these samples (44 patients) were used in the cluster analysis while the evaluation of the association between the epidemiological and DNA methylation data was performed on a subset of 317 of these tumor samples (278 patients). One tumor sample from a recurrent cancer was included in the cluster analysis but not in the association analysis for epidemiological risk factors. The criteria used for subject selection and sample usage are presented in the Table S5.

### DNA preparation

DNA was extracted from tissue samples contained in paraffin blocks. One five- micron section lightly stained with hematoxylin and eosin was prepared from each paraffin block and examined by our study pathologist (G.H. Kang) in order to mark the location of the normal and abnormal tissues (tumor, dysplasia or metaplasia) on the slide. Tissues from these various regions were carefully microdissected from 1–4 serial stained sections and placed into separate microcentrifuge tubes. DNA was extracted by adding 20 µl of lysis buffer (100 mM Tris, 10 mM EDTA Proteinase K 2 mg/ml, tRNA 0.05 mg/ml) to each sample. If the amount of tissue dissected was in excess, the volume of lysis buffer was increased proportionally. Tissues were digested overnight at 50°C in an incubator with continuous shaking. We added 5 µl of water to each tissue lysate and stored the samples at −20°C until bisulfite conversion.

### Bisulfite conversion and DNA methylation analysis

Eighteen microliters of the tissue lysate solution, containing the extracted DNA, were treated with sodium bisulfite as previously described [23]. DNA methylation measurements are reported as PMR (Percent of Methylated Reference) values, as previously described [23]. In order to estimate the number of reactions that can be analyzed for each sample, we tested a small aliquot of the recovered bisulfite-converted DNA by real time PCR using an ALU-based bisulfite specific, methylation-independent control reaction (HB-313 reaction in Table S1). A total of 79 DNA methylation markers were selected for study and analyzed by MethyLight as described previously [24], [25]. Some of these DNA methylation markers have been described in the literature, while others were developed in our own laboratory for known tumor suppressor gene promoters. Several of these markers have previously shown variability in their DNA methylation levels among tumors of a specific type. We exploited this characteristic in order to investigate the potential associations between specific epigenetic changes in tumors and epidemiological risk factors of exposure. A complete list of the markers used is provided in the Tables S1 and S2. The sample and marker utilization in the DNA methylation analysis is depicted in the Figure S1.

### Epidemiological and Clinical Data

Demographic characteristics included age at diagnosis (continuous), race (white/non-white), and gender, obtained routinely from the USC CSP at the time of cancer reporting. A history of personal exposures one year prior to diagnosis were obtained from an in-person structured interview [22] including smoking history, dietary intake, height, body weight at age 20, age 40, and at diagnosis, and history of reflux diseases/symptoms. Smoking history was evaluated in a number of different ways. First, we considered smoking as a categorical variable, classifying individuals as never, former or current smokers. We also evaluated the average number of cigarettes smoked per day, the age when subjects started and stopped smoking, the total years of smoking, and the number of pack-years of cigarettes smoked.

From dietary questionnaires, information on total calories (in kcal), folate acid (in mg/1000 kcal), total fat (in gram/1000 kcal), and dietary fiber (in gram/1000 kcal) was obtained. Information on the following GERD symptoms three or more years prior to diagnosis was obtained: sour stomach, gas pain, heartburn, and swallow symptoms (none, 1, 2, 3 or 4).

Information on tumor characteristics was obtained from the USC CSP. For this analysis, we considered the anatomic site (EAC, GCA and DGA), clinical stage (*in situ* or localized; regional, direct extension, or lymph nodes only; regional, direct extension, and lymph nodes; distant metastases; unstageable), tumor size (in millimeter), and tumor differentiation (well differentiated; moderately differentiated; poorly differentiated; undifferentiated; unknown differentiation). Time of follow-up from diagnosis (in years) was calculated from vital status information obtained from the USC CSP.

### Cluster Analysis

We performed a two-dimensional unsupervised hierarchical cluster analysis of the DNA methylation profiles. Pearson correlations are calculated as pair-wise distance metric, and clusters are combined using the Ward method, which merges clusters that give the minimum increase in error sum of square within cluster [26]. Measures of DNA methylation give a non-negative continuous value reporting the percent of methylated reference (PMR). PMR values are transformed on the natural log scale (ln(PMR+1)) and standardized across gene markers before use in the above cluster analysis.

### Statistical analysis for epidemiological risk factors assessment and clinical outcome

We performed logistic regression using Generalized Estimating Equations (GEE) and robust variance estimates to evaluate the associations between the DNA methylation group variable and all other variables of interest. To evaluate the associations between the number of methylated genes (0–9) and the variables of interest we used a linear link function. A PMR of 10 was used as a cut point for each DNA methylation marker. These approaches allowed us to analyze all tumor samples, accounting for the correlation in outcome among tissue samples obtained from a single patient. P-values were reported both with and without adjusting for covariates. In the analyses with adjustment, we controlled for sex and/or tumor site. Adjusted odds ratios and 95% confidence intervals (CI) were reported.

Patient survival was analyzed using a Cox regression model, with DNA methylation group as a predictor variable. For subjects with more than one tissue sample, the average group value was used as a summary for DNA methylation group membership. Tumor stage, differentiation, site, sex and the number of methylated genes were tested as potential important predictors. For subjects with more than one tissue sample, the average number of methylated genes was used as a substitute for the number of methylated genes. Hazard ratios (HR) with 95% CIs and p-values were calculated and reported for the above continuous and categorical variables. Trend effects for the ordinal categorical variables in relation to survival were also evaluated using ordinal coding for the variables in the Cox regression model. Furthermore, we analyzed the relationship between the summary group variable and survival after adjusting for either tumor stage or tumor differentiation. The association of the summary DNA methylation group and the subjects' survival times were assessed using the log rank test for those in either Group 1 or Group 2. All p-values reported are two-sided and were evaluated at the 0.05 level. We accounted for multiple testing using the Benjamini and Hochberg approach [27].

## Results

### DNA methylation profiles of gastroesophageal tumors

We generated DNA methylation profiles of gastroesophageal tumor tissue samples using a panel of 79 markers (Table S1 and S2) by MethyLight analysis. The samples included three different types of gastroesophageal tumors, EAC, GCA, and DGA. Some of the 79 selected markers have been previously tested on a limited number of EAC tissues [17]. The remaining markers have been reported to be either methylated in other types of cancers, or were recognized for their potential involvement in cancer development (Table S1). Due to the variability in the amounts of DNA obtained from the micro-dissected tissues, the samples were segregated into four groups according to their DNA content (see Figure S1). The group of samples having the highest amount of DNA was analyzed with all 79 markers; the group of samples with the lowest DNA quantity was analyzed with only 9 markers, while those with intermediate DNA quantities were analyzed with either 19 or 39 markers. In total, 318 tissue samples (279 patients) were evaluated for 9 markers, of these, 178 samples (156 patients) were evaluated for an additional 10 markers (19 markers total), 107 (96 patients) were evaluated for an additional 20 markers (39 markers total), and 45 (44 patients) were evaluated for an additional 40 markers (79 markers total) (See Table S5). The choice of DNA methylation markers used for each sample set was based on their degree of variability across various tissue types observed in previous studies [16], [17], [25], [28], [29], and their biological significance.

### Identification of subtype-specific DNA methylation markers and novel DNA methylation subclasses for gastric and esophageal cancers

Out of the 79 DNA methylation markers tested on the 45 tumor samples with the highest amount of DNA available, five markers showed no detectable DNA methylation across all tumors and were excluded from subsequent analysis. We used the remaining 74 informative markers to identify subtype-specific markers and perform cluster analysis for subclass discovery. Using linear regression we found four DNA methylation markers, *MT2*, *SFRP2*, *TFAP2A*, and *TWIST1*, that showed individual association with tumor subtype at p<0.05 significance level, however, after adjusting for multiple comparisons, none of these associations remained statistically significant.

**Figure 1:**
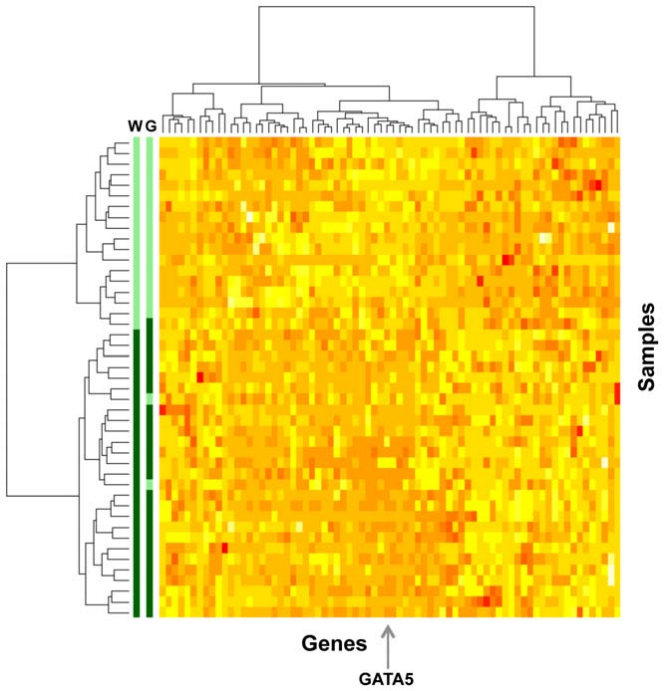
Unsupervised cluster analysis of DNA methylation markers and gastroesophageal adenocarcinimas. Heat map of the standardized (log-transformed percent of methylated reference (PMR) values) DNA methylation values for 74 genes (columns) in 45 gastroesophageal tumor samples (rows) from 44 patients. The clustering was performed using the Ward method. Similar methylation patterns were clustered closely, and two major sample groups were formed based on the DNA methylation profiles of these 74 genes. Based on the Ward method (W, left color bar), the lower 27 samples/27 patients (dark green) were assigned to Group 1, and the upper 18 samples/17 patients (light green) were assigned to Group 2. Based on *GATA5* DNA methylation criterion (G, right color bar), 26 samples/26 patients (dark green) belonged to Group 1, and 19 samples/18 patients (light green) belonged to Group 2. The arrow at the bottom of the diagram shows the *GATA5* DNA methylation column. Red color in the diagram represents high-standardized DNA methylation values. Yellow color in the diagram represents low-standardized DNA methylation values.

In order to summarize the variation in the DNA methylation profiles and to identify new DNA methylation-based tumor subgroups we next performed a two-dimensional unsupervised hierarchical clustering analysis of the same 45 samples and 74 DNA methylation markers. The cluster analysis identified two major sub-groups of gastroesophageal tumors with distinct DNA methylation profiles (Figure 1). There were 27 tumors (27 patients) in the Group 1 and 18 tumors (17 patients) in Group 2. Among the 74 markers, the DNA methylation of the *GATA5* marker showed the highest statistical difference between the two groups (p = 0.005). The ability of *GATA5* marker to distinguish between the two groups was assessed by a Receiver Operating Characteristic (ROC) curve analysis that resulted in an area under the curve (AUC) value of 0.97. To investigate the dependence of these two clusters on *GATA5*, we repeated the clustering excluding *GATA5* from the analysis obtaining a classification accuracy of 93% (42/45) (not shown). This indicates that although *GATA5* is a good representative marker for this classification, it is not solely, or even primarily, responsible for driving the classification.

In order to classify all tumor tissues into the two DNA methylation subgroups, we used logistic regression and the nine markers measured on all 318 tumor tissues to build a classifier for the cluster group outcome. In a model that included *GATA5*, none of the remaining 8 markers added significantly to the prediction of the subgroup (all p>0.05). Using a PMR cut point of 81.4, *GATA5* could predict whether a tumor belonged to Group 1 with 92.6% sensitivity and 94.4% specificity (Figure 1).

### Association between GATA5-based DNA methylation clusters, clinical characteristics, and epidemiological risk factors

We next correlated the nine DNA methylation markers measured on the full set of 317 tumor samples (278 patients) with the clinical and environmental risk variables. Three of the markers, *GATA5*, *ITGA4*, and *RUNX3* were significantly associated with various exposures, such as smoking status (data not shown). However, the associations were no longer significant after adjusting for multiple comparisons. We also did not find any significant associations between the number of methylated genes in the analyzed tumor samples and any of the clinical characteristics or epidemiological risk factors. As an alternative, we then used the two newly identified DNA methylation subgroups to study their association with environmental exposures and clinical outcome. We defined the DNA methylation subgroups using the *GATA5* cut point of 81.4 identified using the 45 tumor samples in the cluster analysis.

Applying this classification criterion to the full set of 317 tumor samples (278 patients, the final classification assigned 186 tumor samples (160 patients) to Group 1 and 131 samples (120 patients) to Group 2. Table 1 shows the characteristics of the two DNA methylation groups.

**Table 1:** Characteristics of DNA Methylation Groups.†

		Group 1	Group 2		
		186 tissues/160 patients	131 tissues/120 patients	p-value1§	Adjusted p-value2*
Mean *GATA5* DNA methylation (SD)		286.9 (235.6)	22.0 (23.1)		
**Subject Characteristics (% of tissues)**					
Sex	Male	161/137 (86.6%)	93/86 (71.0%)	0.003	0.03**
Race	White	141/116 (75.8%)	83/74 (63.4%)	0.03	0.90
Mean age at diagnosis (in years) (SD)		61.3 (8.6)	60.2 (10.9)	0.35	0.79
Mean BMI at diagnosis (in kg/m^2^) (SD)		27.7 (5.2)	25.8 (6.0)	0.045	0.06
Missing (count)		7/7	15/13		
Smoking status				0.003	0.07
Never		41/37 (22.0%)	51/45 (38.9%)		
Ever		145/123 (78.0%)	80/75 (61.1%)		
	Former smokers	95/81 (51.1%)	38/36 (29.0%)		
	Current smokers	50/42 (26.9%)	42/39 (32.1%)		
Mean number of cigarettes per day (SD)		43.3 (33.5)	53.3 (38.8)	0.03	0.14
Total fat (in gram/1000 kcal)		43.1 (7.7)	40.4 (9.1)	0.01	0.26
Presence of symptoms: sour stomach, gas pain, heartburn, and swallow symptoms				0.01	0.07
None		85/74 (48.0%)	77/70 (62.1%)		
One		30/24 (17.0%)	21/19 (16.9%)		
Two		44/37 (24.9%)	13/13 (10.5%)		
Three or four		18/17 (10.2%)	13/12 (10.5%)		
Missing (count)		9/8	7/6		
**Tumor Characteristics (% of tissues)**					
Tumor site/subtype				0.0003	0.003***
Esophageal Adenocarcinoma		73/60 (39.3%)	29/29 (22.1%)		
Gastric Cardia Adenocarcinoma		74/61 (39.8%)	47/38 (35.9%)		
Distal Gastric Adenocarcinoma		39/39 (21.0%)	55/53 (42.0%)		
Tumor stage				0.18	0.28
1) In situ or localized		38/35 (20.4%)	22/20 (16.8%)		
2) Regional, direct extension or lymph nodes only		32/24 (17.2%)	20/18 (15.3%)		
3) Regional, direct extension & lymph nodes		62/53 (33.3%)	34/30 (26.0%)		
4) Distant metastases or unstageable		54/48 (29.0%)	55/52 (42.0%)		
Mean tumor size (mm) (SD)		50.2 (22.5)	52.8 (28.5)	0.51	0.66
Size unknown (count)		58/53	41/41		
Tumor differentiation				0.20	0.23
Well or Moderately differentiated		50/39 (26.9%)	34/32 (25.9%)		
Poorly differentiated		123/108 (66.1%)	79/70 (60.3%)		
Undifferentiated		8/8 (4.3%)	7/7 (5.3%)		
Differentiation unknown		5/5 (2.7%)	11/11 (8.4%)		

†Except for those specified, the information in this table means Count of Tissue Samples/Count of Subjects (% tissues) or Mean (SD).

§p-value1 was computed using GEE and no adjustment variables.

*p-value2 was calculated using GEE after adjusting for tumor site and sex.

**p-value was calculated for the sex variable using GEE after adjusting for tumor site.

***p-value was calculated for the tumor site variable using GEE after adjusting for sex.

There was a difference in the proportion of males between the groups that remained statistically significant even after adjusting for tumor type (p = 0.03). The association with race did not reach statistical significance after sex and tumor type were taken into consideration, and the mean age at the time of diagnosis did not differ by group. BMI at ages 20 and 40 (Table S3) was not associated with the two groups but BMI at the time of diagnosis was borderline significantly associated with DNA methylation group (p = 0.06) after adjusting for tumor type and sex (Table 1).

Smoking status was borderline statistically significantly associated with the DNA methylation group (p = 0.07) after sex and tumor type were taken in consideration (Table 1). The proportion of patients who had ever smoked was higher in Group 1 than in Group 2; this was mainly due to an excess of former smokers (51.1% in Group 1 vs. 29.0% in Group 2 were former smokers). However, there were no significant differences in the average number of cigarettes smoked per day between groups (Table 1) or other smoking parameters (Table S3) after adjusting for sex and tumor type. None of the four dietary variables investigated in this analysis differed significantly between the groups (Table S3). Intake of total fat was significantly lower in Group 2 compared to Group 1 (p = 0.01), but this finding was substantially weakened after sex and tumor type adjustment (p = 0.26) (Table 1). In addition, the history of digestive gastrointestinal symptoms (sour stomach, gas pain, heartburn and swallow symptoms) prior to the diagnosis of cancer was borderline significantly associated with the groups after adjusting for sex and tumor type (Table 1).

Table 1 (bottom half) summarizes the tumor characteristics of the two DNA methylation groups. Tumor site/subtype differed significantly between Group 1 and Group 2 after adjustment for sex (p = 0.003). Esophageal and gastric cardia adenocarcinomas accounted for 39.3% and 39.8%, respectively, of subtypes in Group 1, while 42.0% of samples in Group 2 were distal gastric adenocarcinomas. There were no significant differences in tumor stage, size, or degree of differentiation between groups (all p>0.05).

In the analysis of all tissue samples, only two subjects had tissues discordant for *GATA5* subgroup. A subject-level analysis, classifying subjects into groups based on their average *GATA5* methylation value, showed results similar to those in Table 1 (results not shown). The associations between *GATA5* subgroup and BMI, and *GATA5* subgroup and smoking remained statistically significant even after adjusting for sex and tumor site (p = 0.03 and 0.04 respectively).

The significant associations between the DNA methylation groups and several variables of interest were reflected in the calculated odds ratios (Table S4). More subjects in Group 1 had esophageal or gastric cardia adenocarcinoma than those in Group 2. Males were twice as likely to belong to Group1 than females (95% CI = 1.11–4.03) after controlling for the effect of tumor site, although there were no significant differences in whites versus non-whites. There were twice as many ever smokers in Group 1 than in Group 2 (see Table S4).

### Association between GATA5-based DNA methylation clusters and survival characteristics

**Figure 2:**
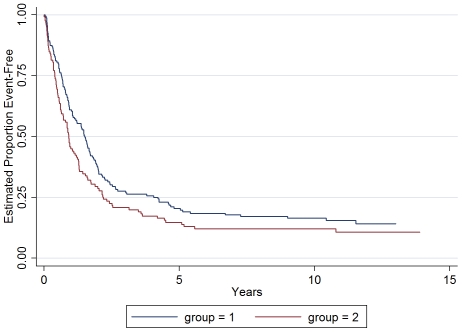
Kaplan Meier Survival Curve of patients with gastroesophageal adenocarcinoma stratified by DNA methylation group. Two subjects with multiple tissue samples that have different DNA methylation group values were included in Group 1.

The median survival time for the patients in Group 1 was longer than those in Group 2 (Table 2). The Kaplan-Meier Survival curves indicated a better overall survival for patients in Group 1(log-rank p = 0.04, Figure 2). However, the stage and the degree of differentiation of the tumors were also independently associated with the survival time (log-rank p<0.0001 and p = 0.01 respectively). The mortality risk of the patients with more advanced stage disease was higher than those with *in situ* or localized tumors, with as much as 6.5-fold higher mortality risk for those with distant metastases or unstageable disease. Compared to patients with well- or moderately-differentiated tumors, patients with undifferentiated tumors had higher rates of death (HR = 1.88, 95% CI = 1.05–3.37). The higher mortality risk assessment of Group 2 patients did not change after adjustment for tumor differentiation (95% CI = 1.14–1.93, p = 0.004). However, after adjusting for tumor stage, the association between DNA methylation subgroup and mortality risk disappeared (stage adjusted HR = 1.2, 95% CI = 0.88–1.5, p = 0.30). Except for the above three variables, we did not find any other statistically significant predictors for patient survival (all p>0.05) (Table 2).

**Table 2:** Association of patient and tumor characteristics with survival time.

Variable	Number of patients (N = 278)	Number of death (N = 239)	Hazard Ratio (95% CI)*	p-value*
DNA methylation Group				0.04
Group 1	160**	135**	1.00	
Group 2	118	104	1.31 (1.01–1.69)	
Sex				0.07
Male	221	198	1.00	
Female	57	41	0.73 (0.52–1.02)	
BMI at diagnosis			0.99 (0.96–1.01)	0.29
Smoking status				0.90
Never	82	70	1.00	
Former	116	102	0.97 (0.72–1.32)	
Current	80	67	1.05 (0.75–1.47)	
Tumor site				0.42
Esophageal Cancer	88	79	1.00	
Gastric Cardia	98	87	1.09 (0.80–1.47)	
Distal Gastric	92	73	0.88 (0.64–1.21)	
Tumor size			1.01 (1.00–1.01)	0.06
Tumor stage				<0.0001
1) In situ or localized	55	36	1.00	
2) Regional, direct extension or lymph nodes only	42	31	1.39 (0.86–2.26)	
3) Regional, direct extension & lymph nodes	82	73	2.48 (1.65–3.73)	
4) Distant metastases or unstageable	99	99	6.54 (4.35–9.86)	
Trend effect on tumor stage			1.93 (1.68–2.21)	<0.0001
Tumor differentiation				0.04
Well- or Moderately-differentiated	71	59	1.00	
Poorly-differentiated	176	154	1.21 (0.90–1.64)	
Undifferentiated	15	14	1.88 (1.05–3.37)	
Differentiation not known†	16	12	0.67 (0.36–1.25)	
Trend effect on tumor differentiation			1.29 (1.00–1.66)	0.05

*Hazard ratios, 95% CIs, and p-values were calculated using Cox regression.

**Two subjects with multiple tissue samples that have different DNA methylation group values were included in Group 1 here. They both died before the end of the study.

†This category was not included in the trend test.

## Discussion

Previous DNA methylation studies of esophageal and gastric cancers indicated that cancer-associated DNA methylation changes could be used as molecular tools to explore and understand the clinical and epidemiological features associated with these diseases. Our present study allowed us to validate the feasibility of conducting population-based studies using DNA methylation analysis on single slides of formalin-fixed paraffin-embedded tissues specimens. These studies are technically challenging due to the large number of samples and markers involved in the analyses, and to issues related to sample integrity associated with the preservation process of archival specimens. The MethyLight technology employed in this study is one of the few available high-throughput DNA methylation assays compatible with automation that can also be applied to single slides of paraffin-embedded tissues. Despite our efforts to include a large number of cases in the DNA methylation analysis, our emphasis in obtaining high-quality data, made it necessary to eliminate many samples, due to the low quality and quantity of the DNA extracted from the paraffin-embedded tissues. However, the final sample set included 278 individuals with information on survival time, 239 of whom had died, providing sufficient numbers of individuals to explore association between DNA methylation and clinical outcome.

We were not able to identify DNA methylation profiles and markers capable of distinguishing between the different subtypes of gastric and esophageal cancers using our collection of 79 reactions. Our finding is supportive of results of a recent study that showed modest differences in DNA methylation between normal gastric cardiac mucosa and esophageal squamous mucosa, despite considerable differences in histology and expression profiles between these tissues [21]. These findings underscore the need of using genome-wide approaches with larger numbers of DNA methylation markers and larger sets of normal and pathological samples in order to achieve this goal.

We successfully identified two new subgroups of tumors with distinct DNA methylation profiles among the esophageal and gastric cardia adenocarcinomas that could be best predicted by DNA methylation of the *GATA5* marker alone. Membership in the two newly identified epigenetic groups was significantly correlated with sex and tumor subtype. Epigenetic Group 1, characterized by higher levels of *GATA5* DNA methylation, consisted mainly of men with esophageal or gastric cardia adenocarcinomas. If gender and tumor type are not taking in consideration, the patients in Group 1 were more likely to be smokers or former smokers, had higher BMI values, and experienced symptoms of gastro-esophageal reflux compared to patients in Group 2. Despite the decrease in significance after adjusting for gender and tumor subtype, our findings still suggest a possible combined effect of tobacco, gastrointestinal fluid, dietary micronutrients alterations or products of fat metabolism to induce specific hypermethylation changes associated with the distinct phenotype of EAC and GCA tumors in Group 1. Moreover, the association between the *GATA5* subgroup and BMI and smoking remained statistically significant even after adjusting for sex and tumor subtype when subjects rather than samples were used in the analysis. Group membership appeared to be also a significant predictor for overall survival, but not after controlling for tumor stage. It is possible that tumor stage (and to a lesser extent tumor differentiation) accounted for most of the differences in the patient survival between groups. However, since these two variables did not differ significantly between groups, the association between the methylation groups and overall survival deserves further investigation in future studies. These two new epigenetic groups would require further confirmation in independent populations.

Abnormal DNA methylation of the *GATA5* gene promoter with subsequent loss of function has been previously reported in several cancers including esophageal, [30], gastric [31], colorectal, pancreatic, lung, and ovarian cancers [31], [32], [33], [34]. The large spectrum of cancers in which *GATA5* DNA methylation has been documented suggests that *GATA5* DNA methylation is not cancer type specific. In our study, *GATA5* DNA methylation, was also not significantly associated with any of the three gastroesophageal tumor subtypes. This conclusion is also in agreement with the observations that *GATA5* DNA methylation is a common marker for both types of esophageal cancers, EAC and esophageal squamous cell carcinoma (ESCC) [30], as well as for three different types of lung cancers that each have distinct cells of origin [33]. These results suggest that high *GATA5* DNA methylation levels may be associated with defects in a specific molecular pathway that is common for cells of different embryological origin. It has also been suggested that DNA methylation-mediated loss of GATA5 function may impede the normal process of differentiation and thus contribute to tumorigenesis [31]. However, our finding showing a lack of association between the *GATA5* methylation or *GATA5* methylation-based groups and the degree of tumor differentiation does not support this hypothesis.

The possible mechanisms linking DNA methylation changes to any of the three exposure risk factors detected in this study are not yet known. Several studies have shown a positive association between smoking and methylation of specific genes in various cancers. Smoking induced DNA damage was suggested to cause abnormal promoter DNA hypermethylation in lung cancer [35]. Interestingly, smoking status was not associated with *GATA5* DNA methylation in a study of lung cancers [33]. However, in the same study it was reported that the length of time a person smoked was significantly associated with *GATA5* DNA methylation, while the former smokers showed a trend towards statistical significance of more frequent GATA5 DNA methylation [33]. Moreover, *GATA5* promoter DNA methylation was shown to be associated with high levels of DNA damage caused by radiation exposure [36]. Our results, together with the observations in lung cancers suggest that high levels of *GATA5* DNA methylation could be linked to molecular pathways involving DNA damage and repair due to exposure to DNA damaging agents such as the carcinogens in tobacco. Also, quitting smoking may not reverse the process. Changes in the tobacco constituents in cigarettes over the years may also explain the higher levels of *GATA5* DNA methylation in former smokers compared to current smokers. Presently, the mechanism targeting *GATA5* for methylation in the context of DNA damage is unclear. It is interesting to note that *GATA5* is one of the genes targeted by the Polycomb group for transcription repression in human embryonic stem cells [3], of which a large majority of genes become abnormally hypermethylated in cancers. The relationship between DNA damage and hypermethylation of genes targeted by Polycomb repression in embryonic stem cells remains to be determined.

A positive association between high BMI and methylation levels of a Line region has been recently reported in head and neck squamous cell carcinoma [37]. The effect of a high BMI on DNA methylation levels may be due to inadequate intake, absorption or metabolism of dietary micronutrients such as folate known to be important for normal DNA methylation [38]. Overweight people have lower levels of multiple micronutrients including folate [39]. However, our study did not reveal any significant differences between groups with respect to the dietary variable analyzed including the folic acid levels in the diet. It is also possible that byproducts of the fat metabolism might have a direct effect on epigenetic state. The effects of a high BMI on DNA methylation may be however indirect, mediated by the gastroesophageal reflux that is more common in obese people due to decrease in lower esophageal sphincter function combined with increased intra-abdominal pressure [40]. GERD is thought to mediate the transformation of squamous esophageal epithelium into Barrett's intestinal metaplasia [41] and of gastric cardia mucosa into cardia intestinal metaplasia (CIM) [42]. Abnormal DNA methylation changes specific for EAC and GCA are present in these early lesions [16]. However, the direct effect of the various components of the refluxed juice or the accompanying inflammatory process on DNA methylation has not yet been thoroughly investigated.

The significance of these new epigenetic subclasses of gastroesophageal cancers and their independent or combined possible association to different life style factors will need to be evaluated in future studies using larger number of samples and a more comprehensive marker selection. Also, other yet unmeasured risk factors such as *Helicobacter pylori* infection that may act as confounders, should be taken in consideration in these studies. The identification of markers specific for these epigenetic subclasses of cancers may also help shed light on the molecular mechanisms leading to their development, and become useful tools for clinicians for early detection, prognosis, and prevention of these types of cancers.

## Supporting Information

Figure S1 **Sample and marker utilization in the DNA methylation analysis.** Forty five tumor samples were analyzed with 79 DNA methylation markers. In addition to these 45 samples, 62 more samples had sufficient DNA to be anylyzed with 39 of the 79 DNA methylation markers. In addition to the 107 samples tested on 39 markers, 71 more samples were anlyzed with 19 of the 39 DNA methylation markers. An additional 140 samples were analyzed with 9 markers of the 19 markers. *GATA5* (in red) was one of the 9 DNA methylation markers tested on all the samples.
(TIF)

Table S1 **MethyLight primers and probes details.**
(DOC)

Table S2 **Additional MethyLight primer and probes details.**
(DOC)

Table S3 **Descriptive statistics of DNA methylation groups.**
(DOC)

Table S4 **Associations of DNA methylation groups vs. exposure factors.**
(DOC)

Table S5 **Sample and subject distribution in the DNA methylation analysis and the statistical analysis.**
(DOC)
