# Supplementary figures and images for: Epigenetic Subgroups of Esophageal and Gastric Adenocarcinoma with Differential GATA5 DNA Methylation Associated with Clinical and Lifestyle Factors
Source: PLoS One. 2011 Oct 20;6(10):e25985. doi: 10.1371/journal.pone.0025985 (PMC3197593; doi:10.1371/journal.pone.0025985)

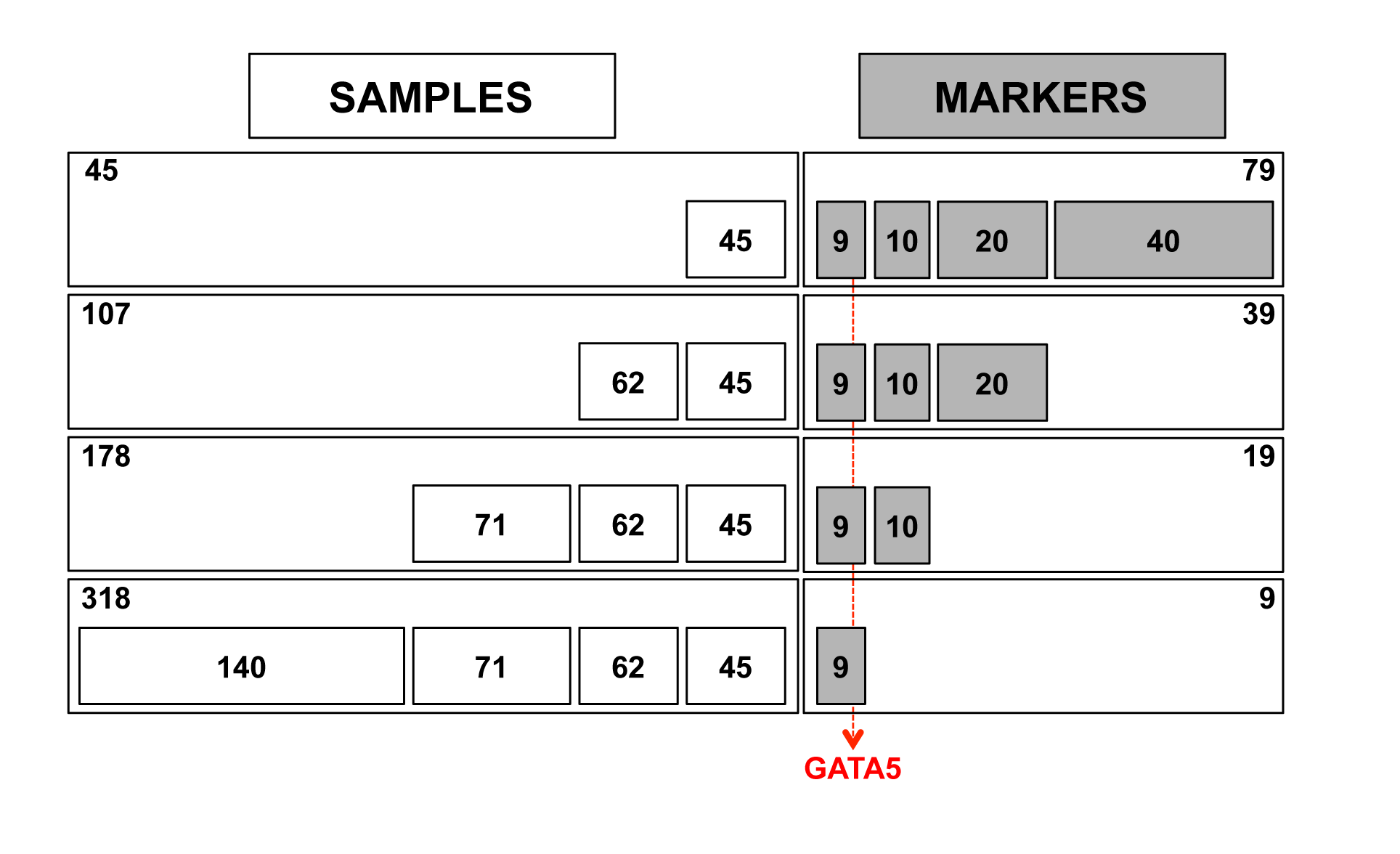

Supplement: Figure S1 — Sample and marker utilization in the DNA methylation analysis. Forty five tumor samples were analyzed with 79 DNA methylation markers. In addition to these 45 samples, 62 more samples had sufficient DNA to be anylyzed with 39 of the 79 DNA methylation markers. In addition to the 107 samples tested on 39 markers, 71 more samples were anlyzed with 19 of the 39 DNA methylation markers. An additional 140 samples were analyzed with 9 markers of the 19 markers. GATA5 (in red) was one of the 9 DNA methylation markers tested on all the samples. (TIF) [file pone.0025985.s001.tif]
