# Supplementary material for: Epigenetic Subgroups of Esophageal and Gastric Adenocarcinoma with Differential GATA5 DNA Methylation Associated with Clinical and Lifestyle Factors
Source: PLoS One. 2011 Oct 20;6(10):e25985. doi: 10.1371/journal.pone.0025985 (PMC3197593; doi:10.1371/journal.pone.0025985)
Supplement: Table S1 — MethyLight primers and probes details. (DOC) [file pone.0025985.s002.doc]

**Table S1. MethyLight primers and probes details**

| **HUGO Gene Nomenclature (if available)** | **Reaction Design Code** | **Source of published reaction** |
| --- | --- | --- |
| ALU | HB-313 | Weisenberger, D.J et al. Nucleic Acids Res 2005;33,:6823-36 |
| ALU | HB-188 | Weisenberger, D.J et al. Nucleic Acids Res 2005;33,:6823-36 |
| APC | HB-153 | Eads CA, et al. Cancer Res 2001;61: 3410-18 |
| BCL2 | HB-140 | Widschwendter M, et al. Cancer Res 2004;64:3807-13 |
| BDNF | HB-258 | Weisenberger DJ, et al. Nat Genet 2006;38:787-93 |
| CACNA1G | HB-158 | Weisenberger DJ, et al. Nat Genet 2006;38:787-93 |
| CACNA1G | HB-162 | Weisenberger DJ, et al. Nat Genet 2006;38:787-93 |
| CADM1 | HB-069 | Described as IGSF4 in Widschwendter M, et al. Cancer Res 2004;64 |
| CALCA | HB-166 | Eads CA, et al. Cancer Res 2001;61: 3410-18 |
| CCND2 | HB-040 | Ehrlich M, et al. Oncogene 2002;21,6694-6702 |
| CDH13 | HB-075 | Weisenberger DJ, et al. Nat Genet 2006;38:787-93 |
| CDKN1A | HB-230 | Weisenberger DJ, et al. Nat Genet 2006;38:787-93 |
| CDKN1C | HB-328 | see Supplemental Table 2 |
| CDKN2A | HB-081 | Eads CA, et al. Cancer Res 2001;61: 3410-18 |
| CGA | HB-237 | Weisenberger DJ, et al. Nat Genet 2006;38:787-93 |
| CHFR | HB-190 | Weisenberger DJ, et al. Nat Genet 2006;38:787-93 |
| COL2A1 | HB-057 | Widschwendter M, et al. Cancer Res 2004;64:3807-13 |
| CRABP1 | HB-197 | Siegmund KD, et al. PLoS One 2007;2:e895 |
| CYP1B1 | HB-078 | Fiegl H, et al. Cancer Epidemiol BioMarkers Prev 2004;13:882-8 |
| DCC | HB-178 | Weisenberger DJ, et al. Nat Genet 2006;38:787-93 |
| DLC1 | HB-218 | Weisenberger DJ, et al. Nat Genet 2006;38:787-93 |
| DLEC1 | HB-225 | Weisenberger DJ, et al. Nat Genet 2006;38:787-93 |
| DRD1 | HB-252 | Weisenberger DJ, et al. Nat Genet 2006;38:787-93 |
| DRD2 | HB-253 | Weisenberger DJ, et al. Nat Genet 2006;38:787-93 |
| EBF3 | HB-229 | Weisenberger DJ, et al. Nat Genet 2006;38:787-93 |
| ESR1 | HB-164 | Eads CA, et al. Cancer Res 2000;60,5021-6 |
| EYA4 | HB-316 | see Supplemental Table 2 |
| GABRA2 | HB-254 | Weisenberger DJ, et al. Nat Genet 2006;38:787-93 |
| GAD1 | HB-255 | see Supplemental Table 2 |
| GATA3 | HB-327 | Weisenberger DJ, et al. Nat Genet 2006;38:787-93 |
| GATA4 | HB-323 | Weisenberger DJ, et al. Nat Genet 2006;38:787-93 |
| GATA5 | HB-326 | Weisenberger DJ, et al. Nat Genet 2006;38:787-93 |
| GDNF | HB-221 | Weisenberger DJ, et al. Nat Genet 2006;38:787-93 |
| GRIN2B | HB-250 | Weisenberger DJ, et al. Nat Genet 2006;38:787-93 |
| HOXA1 | HB-268 | Weisenberger DJ, et al. Nat Genet 2006;38:787-93 |
| IGF2 | HB-319 | Weisenberger DJ, et al. Nat Genet 2006;38:787-93 |
| ITGA4 | HB-321 | Weisenberger DJ, et al. Nat Genet 2006;38:787-93 |
| JUP | HB-203 | Weisenberger DJ, et al. Nat Genet 2006;38:787-93 |
| KL | HB-175 | Weisenberger DJ, et al. Nat Genet 2006;38:787-93 |
| LPHN2 | HB-202 | Weisenberger DJ, et al. Nat Genet 2006;38:787-93 |

**Table S1. MethyLight primers and probes details (continued)**

| **HUGO Gene Nomenclature (if available)** | **Reaction Design Code** | **Source of published reaction** |
| --- | --- | --- |
| LZTS1 | HB-200 | Weisenberger DJ, et al. Nat Genet 2006;38:787-93 |
| MINT/SV2C | HB-161 | Weisenberger DJ, et al. Nat Genet 2006;38:787-93 |
| MINT2 | HB-187 | Weisenberger DJ, et al. Nat Genet 2006;38:787-93 |
| MLH1 | HB-150 | Fiegl H, et al. Cancer Epidemiol BioMarkers Prev 2004;13:882-8s |
| MT1A | HB-205 | Weisenberger DJ, et al. Nat Genet 2006;38:787-93 |
| MT2A | HB-206 | Weisenberger DJ, et al. Nat Genet 2006;38:787-93 |
| MT3 | HB-207 | Weisenberger DJ, et al. Nat Genet 2006;38:787-93 |
| MYOD1 | HB-154 | Eads CA, et al. Cancer Res 2001;61: 3410-18 |
| NEUROD1 | HB-259 | Weisenberger DJ, et al. Nat Genet 2006;38:787-93 |
| NEUROG1 | HB-261 | Weisenberger DJ, et al. Nat Genet 2006;38:787-93 |
| NR3C1 | HB-067 | Weisenberger DJ, et al. Nat Genet 2006;38:787-93 |
| ONECUT2 | HB-242 | Weisenberger DJ, et al. Nat Genet 2006;38:787-93 |
| PENK | HB-163 | Weisenberger DJ, et al. Nat Genet 2006;38:787-93 |
| PGR | HB-149 | Woodson K, et al. Cancer Epidemiol BioMarkers Prev 2005;14:1219-23 |
| PGR | HB-169 | Widschwendter M, et al. Cancer Res 2004;64:3807-13 |
| PITX2 | HB-235 | Weisenberger DJ, et al. Nat Genet 2006;38:787-93 |
| PRKAR1A | HB-214 | Weisenberger DJ, et al. Nat Genet 2006;38:787-93 |
| PYCARD | HB-228 | Weisenberger DJ, et al. Nat Genet 2006;38:787-93 |
| RARRES1 | HB-322 | Weisenberger DJ, et al. Nat Genet 2006;38:787-93 |
| RBP1 | HB-185 | Weisenberger DJ, et al. Nat Genet 2006;38:787-93 |
| RUNX3 | HB-181 | Weisenberger DJ, et al. Nat Genet 2006;38:787-93 |
| SCGB3A1 | HB-194 | Weisenberger DJ, et al. Nat Genet 2006;38:787-93 |
| SERPINB5 | HB-208 | Weisenberger DJ, et al. Nat Genet 2006;38:787-93 |
| SEZ6L | HB-184 | Weisenberger DJ, et al. Nat Genet 2006;38:787-93 |
| SFN | HB-174 | Weisenberger DJ, et al. Nat Genet 2006;38:787-93 |
| SFRP1 | HB-201 | Weisenberger DJ, et al. Nat Genet 2006;38:787-93 |
| SFRP2 | HB-280 | Weisenberger DJ, et al. Nat Genet 2006;38:787-93 |
| SFRP4 | HB-281 | Weisenberger DJ, et al. Nat Genet 2006;38:787-93 |
| SFRP5 | HB-282 | Weisenberger DJ, et al. Nat Genet 2006;38:787-93 |
| SLC6A20 | HB-079 | Weisenberger DJ, et al. Nat Genet 2006;38:787-93 |
| SMAD9 | HB-315 | Weisenberger DJ, et al. Nat Genet 2006;38:787-93 |
| TERT | HB-074 | Fiegl H, et al. Cancer Epidemiol BioMarkers Prev 2004;13:882-8 |
| TFAP2A | HB-314 | Weisenberger DJ, et al. Nat Genet 2006;38:787-93 |
| THRB | HB-216 | Weisenberger DJ, et al. Nat Genet 2006;38:787-93 |
| TIMP3 | HB-167 | Eads CA, et al. Cancer Res 2001;61: 3410-18 |
| TITF1 | HB-213 | Fiegl H, et al. Cancer Epidemiol BioMarkers Prev 2004;13:882-8 |
| TNFRSF10C | HB-308 | Weisenberger DJ, et al. Nat Genet 2006;38:787-93 |
| TP73 | HB-177 | Weisenberger DJ, et al. Nat Genet 2006;38:787-93 |
| TSHR | HB-141 | Weisenberger DJ, et al. Nat Genet 2006;38:787-93 |
| TWIST1 | HB-047 | Muller HM, et al. Cancer Lett 2004;209:231-6 |
