# Supplementary material for: Epigenetic Subgroups of Esophageal and Gastric Adenocarcinoma with Differential GATA5 DNA Methylation Associated with Clinical and Lifestyle Factors
Source: PLoS One. 2011 Oct 20;6(10):e25985. doi: 10.1371/journal.pone.0025985 (PMC3197593; doi:10.1371/journal.pone.0025985)
Supplement: Table S2 — Additional MethyLight primer and probes details. (DOC) [file pone.0025985.s003.doc]

**Table S2.** Additional MethyLight primer and probes details

| **HUGO Gene Nomen-clature (if available)** | **Reaction Design Code** | **Chromosomal Location** | **Forward Primer Sequence** | **Reverse Primer Sequence** | **Probe Oligo Sequence*** |
| --- | --- | --- | --- | --- | --- |
| CDKN1C | HB-328 | 11p15.5 | TTATCGGATAGTTAGGTAGTCGTCGC | AAAACGAAAACCGAACGCAA | 6FAM-CGCGCCGCCCGACTCTACGTAT-BHQ |
| EYA4 | HB-316 | 6q23 | GGAAAGAGTTGCGGGAAAAGT | ACCAAAACTCCGAACTACGACAAA | 6FAM-AACGCGCCCAACCGCCG-BHQ-1 |
| GAD1 | HB-255 | 2q31 | ATTTTTATTAGAGGGCGTTAAGAGTTTAGA | CGCTCGAACGCTAACGAAA | 6FAM-CCCGAAAACGCAAAACCTCTCCGT-BHQ-1 |

***** All primer and probe sequences are listed 5' to 3'. All probes have a 5' 6FAM fluorophore, and a Black Hole Quencher (BHQ-1) at the 3' end.
