# Supplementary material for: Epigenetic Subgroups of Esophageal and Gastric Adenocarcinoma with Differential GATA5 DNA Methylation Associated with Clinical and Lifestyle Factors
Source: PLoS One. 2011 Oct 20;6(10):e25985. doi: 10.1371/journal.pone.0025985 (PMC3197593; doi:10.1371/journal.pone.0025985)
Supplement: Table S3 — Descriptive statistics of DNA methylation groups. (DOC) [file pone.0025985.s004.doc]

**Table S3. Descriptive statistics of DNA methylation g**roups

|  | **Group 1** | **Group 2** |  |
| --- | --- | --- | --- |
|  | **186 tissues/160 patients** | **131 tissues/120 patients** | **p-value*** |
| **Subject Characteristics** |  |  |  |
| BMI at age 20 (in kg/m^2^) | 22.2 (3.2) | 21.7 (3.1) | 0.25 |
| Missing (count) | 14 | 26 |  |
| BMI at age 40 (in kg/m^2^) | 25.7 (4.4) | 24.7 (4.1) | 0.10 |
| Missing (count) | 14 | 26 |  |
| Among smokers |  |  |  |
| Age start smoking | 17.6 (4.9) | 18.6 (5.6) | 0.23 |
| Age stop smoking | 50.6 (12.1) | 52.9 (13.1) | 0.24 |
| Total year of smoking | 33.0 (12.8) | 34.3 (13.8) | 0.50 |
| Number of pack-year | 47.9 (36.2) | 46.0 (37.4) | 0.72 |
| Missing (count) | 1 | 1 |  |
| Total calorie (in kcal) | 2736.1 (1157.4) | 2523.6 (1065.5) | 0.12 |
| Folate acid (in mg/1000 kcal) | 172.6 (55.3) | 180.9 (64.8) | 0.26 |
| Dietary fiber (in gram/1000 kcal) | 10.9 (3.8) | 11.5 (4.5) | 0.22 |

† Except for those specified, Mean (SD) are reported.

* p-values were computed using GEE.

** Observations were deleted when calculating the p-values.
