# Supplementary material for: Epigenetic Subgroups of Esophageal and Gastric Adenocarcinoma with Differential GATA5 DNA Methylation Associated with Clinical and Lifestyle Factors
Source: PLoS One. 2011 Oct 20;6(10):e25985. doi: 10.1371/journal.pone.0025985 (PMC3197593; doi:10.1371/journal.pone.0025985)
Supplement: Table S4 — Associations of DNA methylation groups vs. exposure factors. (DOC) [file pone.0025985.s005.doc]

**Table S4. Associations of DNA methylation groups vs. exposure f**actors

| **Variable** | Adjusted Odds Ratio (95% CI)* | **p-value*** |
| --- | --- | --- |
| Tumor site |  | 0.003*** |
| Esophageal vs. Distal Gastric Adenocarcinoma | 3.02 (1.62-5.65)*** |  |
| Gastric Cardia vs. Distal Gastric Adenocarcinoma | 2.08 (1.14-3.81)*** |  |
| Sex Male vs. Femal | 2.12 (1.11-4.03) ** | 0.03** |
| Race White vs. Non-White | 0.96 (0.49-1.86) | 0.90 |
| BMI at diagnosis (per 5kg/m^2^ increase) | 1.34 (0.98-1.82) | 0.06 |
| Number of cigarettes per day | 0.99 (0.99-1.00) | 0.14 |
| Total fat density | 1.02 (0.99-1.05) | 0.26 |
| Smoking status Ever vs. Never smoker | 1.73 (0.95-3.13) | 0.07 |
| Former vs. Never smoker | 2.26 (1.17-4.38) |  |
| Current vs. Never smoker | 1.27 (0.65-2.49) |  |
| Presence of symptoms: sour stomach, gas pain, heartburn, and swallow symptoms |  | 0.07 |
| One vs. None | 1.18 (0.57-2.45) |  |
| Two vs. None | 2.40 (1.18-4.84) |  |
| Three/Four vs. None | 0.97 (0.39-2.37) |  |

* Odds ratios, 95% CI’s and p-values were calculated for the variables using GEE after adjusting for tumor site and sex.

** Odds ratio, 95% CI and p-value were calculated for the sex variable using GEE after adjusting for tumor site.

*** Odds ratios, 95% CI’s and p-value were calculated for the tumor site variable using GEE after adjusting for sex.
