# Supplementary material for: Epigenetic Subgroups of Esophageal and Gastric Adenocarcinoma with Differential GATA5 DNA Methylation Associated with Clinical and Lifestyle Factors
Source: PLoS One. 2011 Oct 20;6(10):e25985. doi: 10.1371/journal.pone.0025985 (PMC3197593; doi:10.1371/journal.pone.0025985)
Supplement: Table S5 — Sample and subject distribution in the DNA methylation analysis and the statistical analysis. (DOC) [file pone.0025985.s006.doc]

**Table S5.** Sample and subject distribution in the DNA methylation analysis and the statistical analysis

|  | **Number of Subjects (Organ Site)*** | **Number of Tissue Samples (Tissue Type)**** |
| --- | --- | --- |
| **Criteria for Subjects Selection and Sample Usage** |  |  |
| Subjects initially enrolled | **942** |  |
| Subjects that signed release form | **879** |  |
| Subjects with available tumor blocks | **542** |  |
| Subjects with sufficient tissue in the blocks | **380** | **523** |
| Subjects with sufficient tissue on the cut slides | **312** | **440** (332T/83N/25O) |
| **DNA Methylation analysis** |  |  |
| Subjects/Tumor Samples used in the DNA methylation analysis | **279** (89E/98C/92D) | **318^†^** (318T) |
| Subjects/ Tumor Samples tested on 9 DNA methylation markers | **279** (89E/98C/92D) | **318^†^** (318T) |
| Subjects/ Tumor Samples tested on 19 DNA methylation markers | **156** (57E/64C/35D) | **178** (178T) |
| Subjects/ Tumor Samples tested on 39 DNA methylation markers | **96** (32E/44C/20D) | **107** (107T) |
| Subjects/ Tumor Samples tested on 79 DNA methylation markers | **44** (16E/23C/5D) | **45** (45T) |
| **Statistical Analysis** |  |  |
| Subjects/Tumor Samples used for clustering (74 markers) | **44** (16E/23C/5D) | **45^††^** (45T) |
| Subjects/Tumor Samples used for the methylation marker / exposure association analysis (9 markers) | **278** (88E/98C/92D) | **317** (317T) |
| Subjects/Tumor Samples used for GATA5-defined Group1 and 2 / exposure association analysis | **278** (88E/98C/92D) | **317** (317T) |
| Subjects/Tumor Samples used for GATA5-defined Group1 and 2 / survival analysis | **278** (88E/98C/92D) | **317** (317T) |

* E=esophagus, C=cardia stomach, D=distal stomach

** T=tumor, N=normal, O=Metaplasia or Dysplasia

**^†^** One of these samples was excluded from the statistical analysis for exposure

**^††^** One of the samples used in the cluster analysis was not among the 317 tumors used for exposure association analysis
